# Deep-sea microbially influenced corrosion and biomineralization

**DOI:** 10.3389/fmicb.2025.1605909

**Published:** 2025-07-17

**Authors:** Yanchen Ge, Can Wang, Ini-Ibehe Nabuk Etim, Sikandar Khan, Chengpeng Li, Luhua Yang, Jia Liu, Peijia Yi, Jiazhi Liu, Wolfgang Sand, Ruiyong Zhang

**Affiliations:** ^1^State Key Laboratory of Advanced Marine Materials, Institute of Oceanology, Chinese Academy of Sciences, Qingdao, China; ^2^University of Chinese Academy of Sciences, Beijing, China; ^3^Marine Chemistry and Corrosion Research Group, Department of Marine Science, Akwa Ibom State University, Uyo, Nigeria; ^4^Department of Biotechnology, Shaheed Benazir Bhutto University, Sheringal, Pakistan; ^5^Aquatic Biotechnology, University of Duisburg-Essen, Essen, Germany; ^6^Guangxi Key Laboratory of Marine Environmental Science, Institute of Marine Corrosion Protection, Guangxi Academy of Sciences, Nanning, China

**Keywords:** biomineralization, microbially influenced corrosion, deep-sea, interfacial interaction, marine environment

## Abstract

Microbially influenced corrosion (MIC) and biomineralization are widely observed in marine, deep-sea, freshwater, and soil ecosystems. Recently, MIC and biomineralization associated with biofouling have significantly impacted marine resources, including deep-sea minerals and organisms. Notably, uncontrolled biomineralization by certain microorganisms, such as barnacles adhering to ship hulls, can lead to structural damage and economic challenges due to biocorrosion. Biomineralization can be categorized into induced mineralization and controlled mineralization. In natural environments, induced biomineralization is the predominant process. The mechanisms of induced biomineralization and MIC in extreme deep-sea environments have attracted significant attention. The factors influencing these processes are highly complex. The microbial-material interfaces serve as the primary sites for key biochemical reactions driving biocorrosion and biomineralization. Within these interfaces, biofilms, their secreted extracellular polymers, and extracellular electron transfer mechanisms play crucial roles in these processes. Thus, a comprehensive understanding of MIC and biomineralization under deep-sea environmental conditions is essential. Investigating the relationship between these phenomena and exploring their underlying mechanisms are critical for both research advancements and industrial applications.

## 1 Introduction

The deep sea is one of the Earth’s most diverse and species-rich habitats (Saide et al., 2021). As terrestrial resources gradually deplete, there has been an increased effort to explore deep-sea oil and gas, minerals, and biological resources using advanced equipment. However, deep-sea environments pose significant challenges to material durability due to extreme physicochemical conditions such as high hydrostatic pressure, low temperatures, variable salinity, low dissolved oxygen, and fluctuating pH. These factors can significantly accelerate corrosion processes, especially when compounded by microbial activity. Microbiologically influenced corrosion (MIC) and biomineralization are two critical phenomena in deep-sea settings that directly affect the structural integrity of subsea equipment and infrastructure (Wu et al., 2025). Despite technological advancements in deep-sea operations, the role of microorganisms in promoting corrosion or facilitating mineral deposition remains insufficiently understood (Xiao et al., 2025). In particular, the ways in which deep-sea microorganisms survive, adapt, and interact with metal surfaces under such extreme conditions have yet to be fully elucidated. Recent studies have shown that microbial biofilms may enhance localized corrosion or, conversely, induce the formation of protective mineral layers through biomineralization. These processes are thus essential considerations in evaluating material performance and failure mechanisms in deep-sea environments.

Biomineralization processes are widespread in the ocean and play a crucial role in the marine geochemical cycles (He et al., 2023). Biomineralization refers to the differentiation, migration, enrichment, and transformation of inorganic substances under biological influence. It is a process where mineral formation is controlled or influenced by organic matter, converting dissolved ions into inorganic minerals. It occurs through two primary mechanisms: biologically controlled mineralization (BCM), where organisms exert precise control over mineral formation and biologically induced mineralization (BIM), where metabolic byproducts trigger mineralization (Zhang J. et al., 2024). Additionally, Dupraz et al. (2009) introduced biologically influenced mineralization as a third pathway, referring to the passive mineralization of organic matter. These processes not only contribute to the formation of deep-sea minerals, but also have significant engineering applications, including microbial mining and microbial corrosion inhibition (Hamilton, 2003). Biomineralization holds significant importance in various fields. Applications such as self-healing concrete and the fixation of toxic heavy metals have already been widely adopted in aerospace, environmental protection, construction, and energy sectors (Natalio and Maria, 2024; Zhang W. Q. et al., 2023; Park et al., 2022). In addition to biomineralization, MIC, have also been reported to impact the marine environment (Etim et al., 2020; Etim et al., 2018; Lv et al., 2024a).

Microbial activity can alter the processes occurring at the interface, either by accelerating or by slowing down metal corrosion. However, due to the diversity of microorganisms and the immense complexity of their metabolic processes, which are influenced also by environmental factors, MIC still faces challenges in practical applications (Lou et al., 2021a). Early research on MIC focused on the impact of biomineralization processes on metal surfaces and the role of extracellular enzymes within biofilm matrices in influencing electrochemical reactions at the biofilm-metal interface (Beech and Sunner, 2004). Studies suggest that biomineralization plays a key role in the formation of corrosion products through complex electron transfer interactions between microorganisms and metals (Duan et al., 2006). The interactions between biofilms and metal surfaces can lead to MIC, for example, through biomineralization driven by microbial metabolism or by biofilms serving as nucleation sites for mineral precipitation. The layer of corrosion products that forms on a material’s surface is often referred to as a biomineralization film (Borets'ka and Kozlova, 2010; Liu et al., 2022). Many microorganisms exhibit both corrosive and mineralizing properties and their interaction with the same material substrate can result in either mineralization or corrosion, depending on environmental conditions. The dual effect can lead to either corrosion inhibition or corrosion promotion. Understanding the transition from biofilm formation to biomineralization film is crucial for advancing interface studies. Biomineralization can also influence corrosion, providing niches for corrosive microorganisms (Martinez et al., 2022).

Therefore, clarifying the relationship between deep-sea biomineralization and MIC, specifically the mechanisms of corrosion and mineralization, and elucidating microbial-material interfacial corrosion processes from a biomineralization perspective will enhance our understanding of marine and deep-sea environments. This review explores various approaches to studying and improving biogeochemical cycling mechanisms while highlighting how biomineralization in marine environments can contribute to efficient corrosion inhibition and the reduction of corrosion-related losses.

## 2 Deep-sea environment and microbial mineral types

### 2.1 Deep-sea environment and related microorganisms

**Figure 1:**
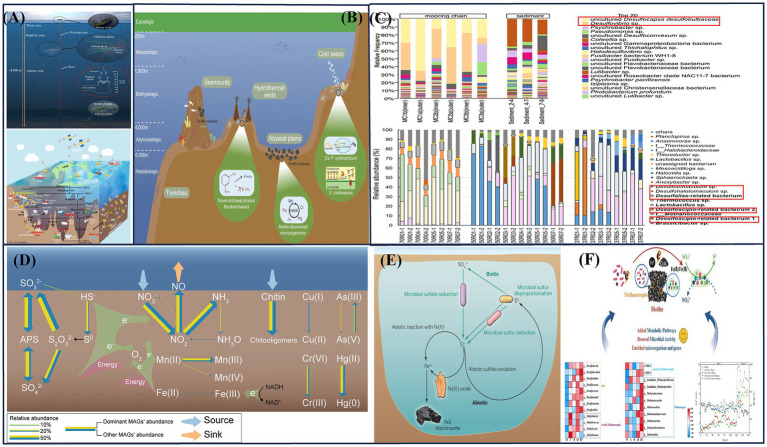
**(A)** Deep-sea service equipment (Feng et al., 2022; Drazen et al., 2020); **(B)** Deep-sea ecosystem (Zhang C. et al., 2024); **(C)** Deep-sea corrosive biotic community (Wakai et al., 2024); (Rajala et al., 2022a); **(D)** Deep sea iron manganese sulfur cycle diagram (Zhang D. et al., 2023); **(E)** Schematic diagram of iron sulfur coupling (Friedrich and Finster, 2014); **(F)** Schematic diagram of methane cycle and sulfur cycle (Liu et al., 2025).

The term deep-sea generally refers to oceans at depths below 200 meters (Danovaro et al., 2020; Li et al., 2023). The deep-sea ecosystem includes hydrothermal vents, cold seeps, whale fall ecosystems, seamounts, oceanic trench ecosystems and abyssal plain (Feng et al., 2022; Simon-Lledó et al., 2023). It also includes incredible volumes of deep-sea water column, as shown in Figure 1B. It is characterized by perpetual darkness, temperatures ranging from −1.0 to 4.0°C or up to 400°C in hydrothermal vents, high pressure, and oligotrophic conditions, making it an extreme environment (Emery, 2001; Feng et al., 2022). Extreme environments are typically characterized by factors such as temperature, pH, pressure, salinity, toxicity, and radiation levels. These environments support a diverse range of microorganisms, known as extremophiles, that have adapted to survive under such conditions (Li et al., 2013). Compared to more familiar environments such as the atmosphere or shallow marine settings, the deep-sea environment has significantly reduced dissolved oxygen levels, which alters corrosion dynamics. Thus, conventional corrosion protection methods are often inadequate to meet the protective requirements of deep-sea equipment (Duan et al., 2023). Figure 1A illustrates representative deep-sea operational equipment, including exploration tools such as remotely operated vehicles and manned submersibles, as well as subsea engineering systems for underwater operations, deep-sea oil and gas production, and mineral resource extraction (Muñoz-Royo et al., 2021; Chen et al., 2024).

Microorganisms in deep-sea environments are highly specialized and form the foundation of biogeochemical and energy cycles, making them indispensable to deep-sea ecosystems. Exploring the different types of microorganisms in various deep-sea regions, along with their physiological and metabolic characteristics, can provide a theoretical foundation for investigating the corresponding corrosion and mineralization mechanisms. These microorganisms include thermophiles, psychrophiles, acidophiles, alkaliphiles, halophiles, and piezophiles, each adapted to extreme environmental conditions. The different microbial types detected in extreme deep-sea environments are shown in Table 1. Since more than 99% of these microorganisms lack closely related described species, significant challenges remain in understanding their diversity, physiological functional traits, and biogeochemical roles. The establishment of a deep-sea microbial library related to corrosion still requires extensive corrosion coupon experiments. Based on a large amount of genomic data, species-corrosion patterns can be derived. Researchers have successfully discovered, isolated, and cultured microbial species from deep-sea hydrothermal vents, cold seeps, marine sediments, deep-sea water, and oceanic crust. These microorganisms primarily include marine bacteria, archaea, and viruses. Among them, sulfate-reducing prokaryotes (SRP) and iron-oxidizing prokaryotes (IOP) are the most commonly associated with corrosion processes (Barton and Northup, 2007). Guezennec et al. (1998) deployed various materials (such as 316 L stainless steel, titanium, copper-nickel alloy, etc.) in the deep-sea hydrothermal vent area of the Mid-Atlantic Ridge. After 4 days of exposure, a significant accumulation of biomass was observed on all material substrates, with the formation of thick but loosely attached biofilms on the surfaces. Analysis of their lipid extracts showed characteristics of SRP. Rajala et al. (2022a) reported an integrative study of the biofilms growing on the surface of corroding mooring chain links that had been deployed for 10 years at about 2 km depth. Wakai et al. (2024) collected slime-like precipitates composed of corrosion products and microbial communities from a geochemical reactor set on an artificial hydrothermal vent for 14.5 months, and conducted culture-dependent and -independent microbial community analyses with corrosive activity measurements, as shown in Figure 1C. The performance of sulfur-metabolizing microorganisms was prominent. Fe-S-driven element coupling has also become a fundamental basis for investigating corrosion mechanisms, as shown in Figure 1E. The methane cycle in marine sediments was closely linked with the sulfur cycle, as shown in Figure 1F (Friedrich and Finster, 2014). Microorganisms in hydrothermal vent systems are predominantly extremophilic bacteria or archaea, which utilize the abundant sulfides near hydrothermal vents to produce biomass. They form the foundation of the entire hydrothermal vent ecosystem. The deep-sea hydrothermal vent regions exhibit a high microbial diversity due to the chemical complexity and rapid environmental fluctuations in these habitats. In contrast, cold seep systems have higher biomass but lower biodiversity, with archaeal communities being the dominant microbial inhabitants. In deep-sea sediments, microbial abundance is influenced by the organic matter content and proximity to continental plates, with heterotrophic microorganisms prevailing in these regions. The oceanic crust, primarily composed of mafic and ultramafic rocks rich in minerals, hosts chemoautotrophic microorganisms that play a key role in biogeochemical cycles, such as those involving Fe, Mn, and S (Wang et al., 2013). For example, the Figure 1D illustrates a schematic of a microbial-dominated ecological function study in a deep-sea ferromanganese nodule deposition area (Zhang D. et al., 2023).

**Table 1:** Microorganisms adapted to extreme deep-sea environments.

Types of microorganisms	Main species
Piezophilic	Bacteria mainly belong to the *γ-proteobacteria* group, including the genera *Photobacterium*, *Shewanella*, *Colwellia*, *Psychromonas*, *Moritella*, and *Thioprofundum*, as well as certain *α-proteobacteria* and *δ-proteobacteria* group Archaea primarily originate from the genera *Thermococcus*, *Pyrococcus*, and *Methanococcus* (Li et al., 2013)
Acidophilic	Archaeon *Aciduliprofundum* boone (Reysenbach et al., 2006)
Thermophilic	*Aquificales*, *Epsilonproteobacteria* subclass, *Thermales* order, *Thermodesulfobacteriaceae*. *Desulfurobacteriaceae*, *Thermaceae* families, and the newly described bacterial phylum represented by the genus *Caldithrix* (Miroshnichenko and Bonch-Osmolovskaya, 2006)
Psychrophilic	*Paracoccus*, *Pseudomonas*, *Halomonas*, *Pseudoalteromonas* (Zeng and Zhao, 2002)

### 2.2 Deep-sea minerals and biomineralization layers

Deep-sea minerals encompass a variety of mineral resources located on the seafloor, primarily distributed across seamounts, ridges, and basins. Common deep-sea mineral deposits include manganese nodules, seafloor cobalt crusts, and hydrothermal deposits, which polymetallic resources formed naturally under deep-sea conditions. Interestingly, recent research suggests that metal nodules in deep sea may have the potential to produce oxygen (Sweetman et al., 2024). Oceanic ferromanganese nodules, a significant underwater metal resource, are widely distributed on the modern seafloor and are rich in Fe, Mn, Ni, Cu, Co, Mo, Li, and rare earth elements. These nodules generally have a core surrounded by concentric mineral layers and are typically found at depths greater than 4,000 m. The primary minerals include Fe-rich birnessite, amorphous ferrihydrite, and various detrital minerals like magnetite, hematite, quartz, calcite feldspar, clay minerals and mica (Hein et al., 2013).

Minerals influence many important microbial activities, including energy production, nutrient acquisition, cell adhesion, and biofilm formation (Hochella, 2002). For example FeMn crust and phosphorite substrates in the Southern California Borderland support enhanced trophic biodiversity and specific trophic niches (Pereira et al., 2024). Interestingly, a recent study found that minerals not only serve as a nutritional source for microorganisms but also act as signaling molecules that regulate microbial adaptation to the environment. Minerals function as “information transmitters” in ecosystems, and the rational use of mineral signals may become a new direction (Zhang et al., 2025).

Most organic carbon mineralization in marine sediments is associated with dissimilatory sulfate reduction (DSR), as shown in Figure 1D (Qian et al., 2019). A wide range of marine organisms and microorganisms have been identified for their role in mineralization. Examples include marine organisms such as sponges and tunicates as well as marine microorganisms that facilitate the mineralization of elements like Fe, S, Mg, Ca, Mn, and Cu, and U. Since these elements exist in multiple valence states, the mineralization layers formed under different environmental and biological conditions exhibit significant diversity.

## 3 Mechanisms and factors influencing biomineralization

**Figure 2:**
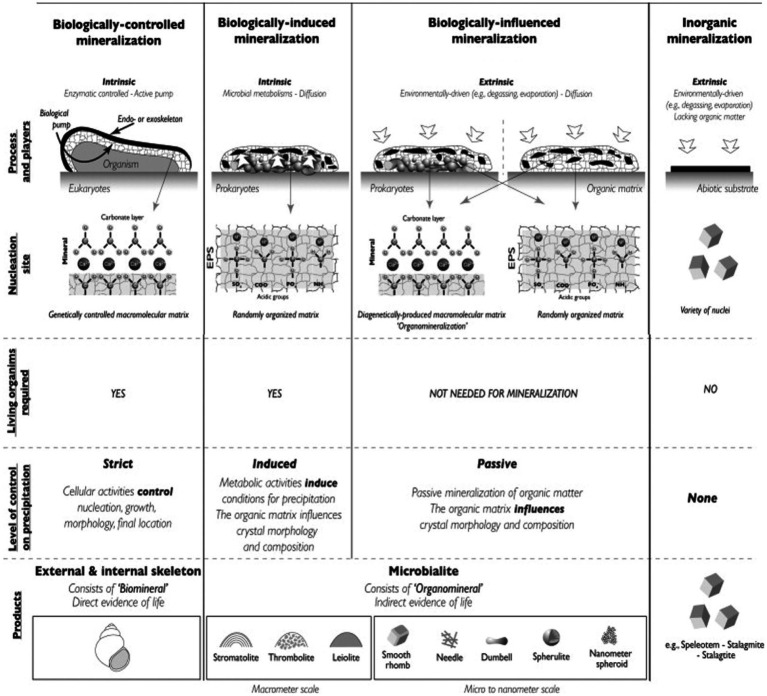
Different types of mineralization associated with both biological and abiotic factors (Dupraz et al., 2009).

Mineralization refers to the process by which microorganisms degrade organic matter into CO_2_, H_2_O, and inorganic substances. As mentioned in the introduction, biomineralization specifically involves microbial generation of minerals through BCM, BIM and biologically influenced mineralization pathways, each with distinct mechanisms. Figure 2 illustrates the different types of mineralization associated with both biological and abiotic factors. Most microbial mineralization processes can induce mineral formation (Dupraz et al., 2009; Weiner and Dove, 2003). Notably, studies have reported that Pd ions on filamentous viruses can undergo spontaneous biomineralization under environmental conditions, forming ligand-free Pd nanowires (Kim et al., 2017). In marine and deep-sea environments, there is growing interest in the formation of minerals such as metal sulfides, sulfate minerals and carbonate minerals. Some of the key biomineralization studies are summarized in Table 2.

**Table 2:** Biomineralization studies.

Minerals	Mineralization layer	Strain/organism	Strain origin	BCM/BIM	References
Metal sulfides	CuS	*Desulphamplus magneticvallimortis* BW-1		BCM	Park et al. (2022)
	CdS	*Pseudomonas stutzeri* 273	Sediments in the East China Sea		Ma et al. (2021a)
		*Idiomarina*sp. OT37-5b	Hydrothermal vent		Ma et al. (2021b)
	ZnS (sphalerite)	SRP, etc.	Seafloor massive sulfide deposits		Hu et al. (2019)
		SRP *Desulfobacteriaceae*	A flooded tunnel within carbonate rocks that host the Piquette Pb-Zn deposit	BIM	Labrenz et al. (2000)
	FeS_2_ (pyrite)	*Desulfovibrio desulfuricans* DSM642		BIM	Duverger et al. (2020)
	Fe_3_S_4_ (greigite)_,_ FeS_2_	a magnetotactic bacterium	Sediment and water from marine and brackish	BCM	Mann et al. (1990)
	Fe_3_S_4_	*Candidatus* Magnetomorum HK-1	Coastal tidal sand flats	BCM	Kolinko et al. (2014)
	FeS (mackinawite), FeS_2_, Fe_3_S_4_	Barnacle	The Yangtze Estuary II shipwreck		Zhao et al. (2024)
Sulfate minerals	Fe_2_(SO_4_)_3_·xH_2_O	Sulfate oxidizing prokaryotes, etc.	Black smoker hydrothermal fragments collected from seamount sites	BIM	Verati et al. (1999)
	FeSO_4_				
	KFe_3_(SO_4_)_2_(OH)_6_				
Carbonate minerals	CaCO_3_ (calcite, aragonite)	Sponge	Offshore and sub-intertidal area		Abbas and Mahmoud (2022)
		Coccolithophore	Crusts from the Magellan Seamount cluster	BIM	Wang et al. (2009)
		*Exiguobacterium mexicanum*	Marine	BIM	Bansal et al. (2016)
	CdCO_3_ (otavite)	Burkholderia-Caballeronia-Paraburkholderia	Sediments of deep-sea hydrothermal vents	BIM	Ma et al. (2024)
	FeCO_3_ (siderite)	*Shewanella oneidensis* MR-4	The Black Sea	BIM	Han X. H. et al. (2024)
	CaMg(CO_3_)_2_	*Bacillus subtilis*	Marine	BIM	Shen et al. (2020)
Metal oxides	Fe_3_O_4_ (magnetite)	*Anoxybacter fermentans* DY22613^T^	Deep-sea hydrothermal sulfide deposit		Li et al. (2019)
	MnO_2_	Mn-oxidizing bacteria	The South China Sea	BIM	Zhao M. et al. (2023)
Phosphate minerals	Fe_3_(PO_4_)_2_·8H_2_O (vivianite)	S - disproportionating *δ-proteobacteria*	Deep basin sediments		Dijkstra et al. (2014)
	NH_4_MgPO_4_·6H_2_O (struvite)	*Alteromonas* (*A.*) *alteriprofundi* HHU 13199^T^, *A*. *alterisediminis* N102^T^	Marine sediment from the South China Sea, deep-sea sediment of the New Britain Trench	BIM	He et al. (2023)
Others	Silver nanoparticles	*Bacillus sp*. VITSSN01	Marine sediment	BIM	Revathy et al. (2014)
	Si-rich nanoparticulate	ANME-SRP	Seafloor methane seep sites		Osorio-Rodriguez et al. (2023)
	ZVI, titanomagnetite	*Methanosarcina barkeri*		BIM	Shang et al. (2020)

### 3.1 Biologically induced mineralization

**Figure 3:**
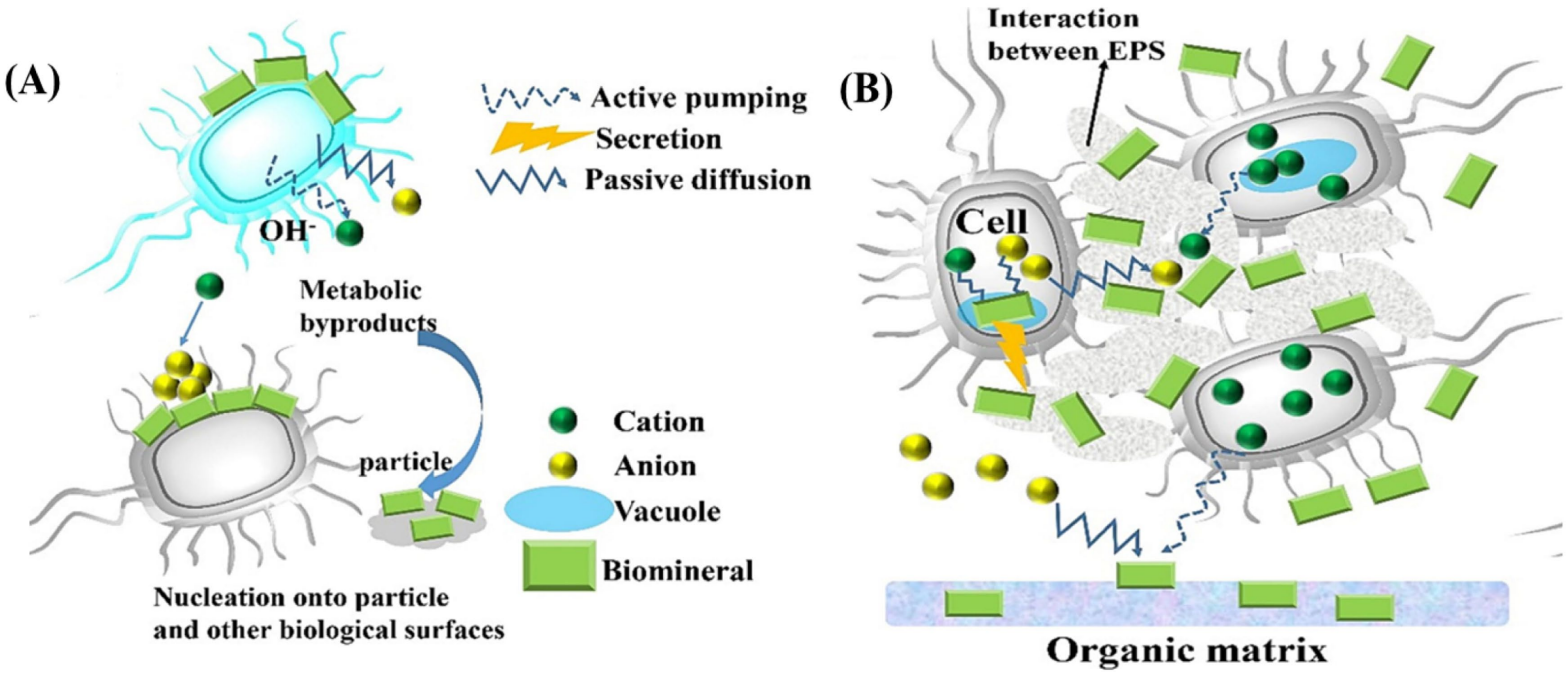
**(A)** Schematic of BIM; **(B)** Schematics of biologically controlled intracellular mineralization shows that nucleation occurs within the cell in a specialized vesicle (Zhang J. et al., 2024).

BIM is a process in which organisms modify their local microenvironment through metabolic activities, creating physicochemical conditions that favor mineral precipitation. However, the resulting products often lack specific biological functions, as illustrated in Figures 2, 3A. BIM is widespread in nature. For example, microbial mineralization contributes to the formation of carbonates, moon milk, silicates, clays, iron and manganese oxides, sulfur, and saltpeter in caves (Northup and Lavoie, 2001). Some microorganisms can directly or indirectly induce the decomposition, hydration, dissolution, or formation of secondary minerals (Banfield et al., 1999). Biomineralization is a two-step process: initially, metal ions electrostatically bind to the anionic surfaces of the cell wall and the surrounding EPS, which then as nucleation sites for crystal growth (Konhauser, 1998). Induced mineralization involves interactions between bacterial surfaces and metal ions in colloidal ionic form. Specific organic and inorganic interactions allow biomineralization to play a potential role in forming high-grade mineral deposits (Leguey et al., 2010). Further studies by He et al. (2023) on deep-sea strains Alteromonas alteriprofundi HHU 13199^T^ and Alteromonas alterisediminis N102^T^ have provided insights into the primary mechanism underlying BIM.

Certain microorganisms, such as SRP, are well known for their role in biomineralization. SRP are among the primary strains studied in marine MIC and biomineralization research. For example, discovered study found that dissimilatory SRP, particularly those from the genera Desulfovibrio and Desulfotomaculum, could deposit electron dense FeS particles within their cells when cultured in an Fe rich medium (Jones et al., 1976). In modern sedimentary environments, SRP are the main producers of sulfides. The mineral composition resulting from SRP metabolic activity is highly dependent on the source of iron. Microorganisms play a key role in the formation of iron sulfides, particularly pyrite (FeS_2_) (Duverger et al., 2020). The application of BIM in engineering, such as self-healing concrete and nanoparticle synthesis, has become a significant research focus.

Microbially-induced calcium carbonate precipitation (MICP) is a naturally occurring process that has gained considerable attention in recent decades (Li et al., 2024). BIM, primarily observed in various Bacillus strains, plays a key role in concrete self-healing. Among them, *Bacillus pasteurii*’s demonstrated excellent mineralization capability, making it a focal point of interest in geotechnical engineering (Debnath and Sen, 2024; Natalio and Maria, 2024). Recently, filamentous fungi have emerged as promising candidates for biomineralization-based self-healing concrete. Their hyphae networks form an interwoven three-dimensional structure, providing nucleation sites for CaCO_3_ precipitation to repair cracks (Van Wylick et al., 2021). Recent advances in fungal-mediated self-healing concrete have further confirmed their potential in sustainable construction materials. Beyond construction applications, biomineralization has significant implications for nanoparticle synthesis. The interactions between proteins and nanoparticles at mineral interfaces have garnered significant attention in the development of novel biomaterial. However, the precise role of proteins in microbial transformation and BIM remains unclear (Liu et al., 2021). Park et al. (2022) described that periplasmic proteins, such as DegP-like proteins and heavy metal-binding proteins, may be involved in the biomineralization process. Additionally, Mn has recently been identified as an accelerator of nitrogen metabolism. Biomineralized MnOx is now believed to mediate ammonia oxidation, offering a potential alternative to conventional nitrogen removal processes (Liu et al., 2024).

### 3.2 Biologically controlled mineralization

BCM is a process in which biological macromolecules and cells actively regulate the reaction of ingested metal ions and anions through various physicochemical mechanisms, leading to the formations of biominerals with specific assembly forms and advanced structures (Weiner and Dove, 2003). The resulting mineralized products often provide organism with specific physiological functions. The BCM process can occur extracellularly, intercellularly, or intracellularly, with the mineralization sites depending on the specific cells involved in the process (Weiner and Dove, 2003). Figure 3B demonstrates an example of intracellular BCM. Almost all BCM processes take place within isolated microenvironment. During mineralization, vesicles often serve as confined microenvironment, guiding biominerals nucleation within microorganisms while controlling the composition and morphology of the resulting biominerals.

A typical representative of BCM is magnetotactic bacteria (MTB), which are Gram-negative bacteria capable of moving along magnetic field lines and oxygen concentration gradients. They are primarily isolated from hypoxic conditions in aquatic niches and rarely from soil (Mistry et al., 2024). MTB can synthesize intracellular nanoscale minerals of magnetite (Fe_3_O_4_) and/or greigite (Fe_3_S_4_), forming magnetosomes that enable magnetotactic orientation. As a result, they serve as an excellent model system for studying biomineralization (Lin et al., 2014). Magnetosomes are generally 20–120 nm in length, consist of highly pure magnetite or greigite and are arranged in chains within MTB. These structures have been regarded as magnetic fossils of life, serving as important clues for exploring early earth conditions and potential extraterrestrial life activities. In some marine protozoa, magnetotactic ability is acquired through the uptake of extracellular symbiotic bacteria, Wherein the protozoa ingest MTB along with their magnetosome chains, internalizing the bacteria (Mandal, 2023). Metal sulfides are a common type of extracellular bacterial biominerals, but intracellular biomineralization has also been observed, such as the formation of greigite in MTB (Park et al., 2022). Numerous MTBs may significantly influence the iron cycle in oxic-anoxic transition zones and anoxic zones, facilitating the deposition of fine-grained magnetite and greigite. Consequently, they enhance the remanent magnetization of sediments (Bazylizinki et al., 1993).

### 3.3 Biologically influenced mineralization

Biologically influenced mineralization is a passive process that does not directly result from microbial activities but rather from interactions between extracellular biopolymers and the geochemical environment (Decho, 2010). In biologically influenced mineralization, external, environmental factors, such as increased alkalinity, play a crucial role in creating conditions favorable for mineral precipitation, rather than microbial metabolism itself. However, an organic matrix is involved in biologically influenced mineralization, influencing the morphology and composition of the resulting crystals. This occurs through interactions between the forming mineral and the organic matter, which serves as a template for precipitation (Dupraz et al., 2009).

### 3.4 Factors influencing biomineralization

#### 3.4.1 EPS and biofilm

The 1988 glossary of the Berlin Dahlem Conference on biofilm structure and function described EPS as “microbially derived organic polymers,” responsible for binding cells and particulate matter together in biofilm systems. Polysaccharides, proteins, nucleic acids, and lipids are the primary components of EPS. The EPS matrix plays a crucial role in biofilms by keeping bacteria in proximity, expanding interactions, capturing and enriching metal ions, and enhancing mechanical strength. Additionally, EPS contributes to biofilm resilience by increasing resistance to antimicrobials and environmental stresses (Flemming, 2016). For example, spores coated with EPS have been shown to enhance biomineralization capabilities (Debnath and Sen, 2024).

Biofilm is an aggregation of microorganisms, in which cells are typically embedded in a self-produced EPS matrix and attached to surfaces (Vert et al., 2012). Bacterial biofilms are formed through a complex yet orderly process involving intra- or inter-cellular communication, environmentally responsive gene expression, and secretion of EPS (Fang et al., 2020). Cultivable microbial populations are more abundant in biofilm samples than in those exposed to natural weathering (Zhang Y. et al., 2023). Bacteria with strong biofilm-forming abilities can adhere to the surfaces, such as carbon steel, forming dense biofilms that may induce biomineralization (Hu et al., 2023). In marine environments, biofilms, along with iron and manganese oxides, are closely associated with biomineralization and biocorrosion caused by iron bacteria in marine environments (Zhang K. et al., 2024). Shift in bacterial populations, including SRP, denitrifying bacteria, and iron-reducing bacteria, are observed within biofilms. Notably, various exogenous factors, such as riboflavin and quorum-sensing compounds, including autoinducer-1 [acyl-homoserine lactone (AHL)] and autoinducer-2, can influence biofilm formation, ultimately affecting mineralization processes (Hao et al., 2024; Zhao J. et al., 2023; Mukherjee and Bossier, 2019).

#### 3.4.2 Biochemical parameters

Biochemical parameters significantly influence microbial community distribution and biomineralization capacity (Zhang Y. et al., 2023). These parameters include pH, temperature, humidity, cell concentration, and enzyme activities. It is generally believed that the morphology of mature magnetite crystals formed by MTB is largely unaffected by environmental conditions. However, Moisescu et al. (2014) reviewed various studies and found that changes in environmental factors such as temperature, pH, external iron concentration, external magnetic field, static or dynamic fluid conditions, and nutrient availability or concentration could affect the biomineralization processes. Research indicates that pressure release, temperature reduction, and alkali metal loss can drive carbonated rare-earth mineralization. Additionally, the coupling of alkali metals and sulfates under high-temperature and high-pressure conditions promotes the formation of sulfate-enriched hydrothermal fluids, which efficiently transport rare-earth elements (Wan et al., 2023).

#### 3.4.3 Metal ions concentration and composition

EPS readily binds various metal ions under certain chemical conditions, facilitating minerals formation. Various studies indicate that metal binding and biomineralization in nature may be influenced directly, through microbial type, energy metabolism, and variations in cell surface and EPS chemistry, and indirectly, via microbial impacts on solution Eh and pH (McLean et al., 1996). For example, Mo, a commonly used metal in steel, has been studied using low-alloyed steels with varying Mo concentrations. It was found that a low concentration of Mo (e.g., 0.6 wt.%) significantly enhanced biofilm formation and mineralization. Mechanistically, Mo ions have been demonstrated to act as chemoattractants for *Pseudoalteromonas lipolytica*, by activating chemotactic pathways (Guo et al., 2019).

#### 3.4.4 Other factors

Other influencing factors include pressure, magnetic fields, and voltage. Debnath and Sen (2024) demonstrated that under pressure conditions, the biomineralization rate of encapsulated microorganisms increased. Similarly, Nan and Pei (2006) produced titanium oxide films under an applied voltage of 250–550 V and found that the biomineralization rate increased with higher voltage resulting in morphological differences among the samples. The structure and composition of these films significantly influenced their biomineralization behavior. Additionally, Liu H. et al. (2016) investigated the effects of magnetic fields (MF) on the biomineralization and corrosion behavior of Q235 steel. Their findings revealed that MF altered the morphology of the biomineralized films, leading to the formation of denser mineralized films.

## 4 MIC and microbe-material interfacial interactions

In deep-sea sediments and similar environments, microorganisms (including bacteria, archaea, fungi, and protozoa) can accelerate corrosion even under conditions typically unfavorable to the process (Rajala et al., 2022b). Understanding biomineralization and MIC processes requires a detailed examination of the interaction between microorganisms and materials. The uniqueness of bacteria and the electrical structure of mineral surfaces impact important processes (Conners et al., 2022). The interactions between biofilms and metal surfaces can lead to two biologically induced material degradation processes: MIC and bioleaching (Parra et al., 2021). Biofilm is a dynamic process, and chemical changes at the metal-biofilm interface can trigger corrosion (Knisz et al., 2023; Anusha and Mulky, 2023; Etim et al., 2025).

### 4.1 Overview of MIC mechanisms

**Figure 4:**
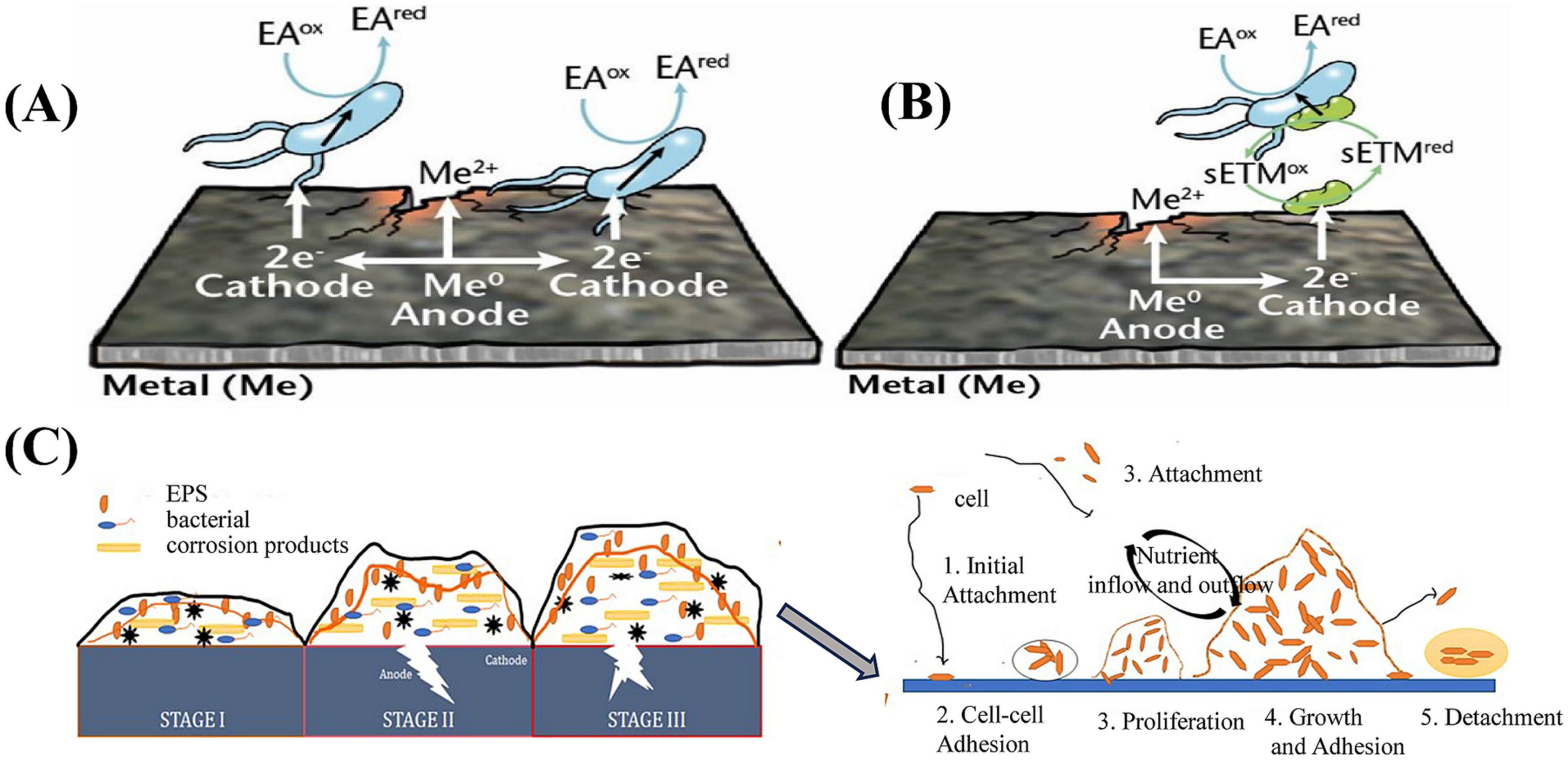
**(A)** Direct EMIC of metals; **(B)** Indirect EMIC of metals, EA: electron acceptor, ED: electron donor; ox: oxidized; red: reduced; sETM: soluble electron transfer mediator (Knisz et al., 2023); **(C)** Biofilm stages and the five-step model of its development (Anusha and Mulky, 2023).

Knisz et al. (2023) described the primary mechanisms of MIC, including oxygen gradient corrosion under sediments, crevice corrosion, metabolite-induced corrosion, and electrochemical microbially influenced corrosion (EMIC). EMIC is classified into two types: direct and indirect. Direct EMIC occurs when microorganisms facilitate extracellular electron transfer (EET) by directly contacting metal surfaces leading to corrosion. Indirect EMIC involves the release of soluble electron mediators by microbes, which act as electron transfer shuttle, and accelerate corrosion of metals, as shown in Figures 4A,B. Biofilms further create additional crevices for various corrosion-related bacteria. Microbial activities within a biofilm can also enhance the influence of abiotic metallurgical conditions, promoting localized corrosion. During pipeline operations, factors such as pressure, stray currents, cathodic protection, and environmental variations can interact synergistically with microorganisms. These combined factors can lead to microbial-induced stress corrosion cracking (SCC), microbially-induced hydrogen-induced cracking (HIC), or crevice corrosion and under-deposit corrosion, where microbially-induced biomineralization occurs at specific locations. Such processes pose a severe risk to pipeline operational safety (Xiong et al., 2018).

### 4.2 Microbe-material (mineral) interfacial interactions

Interfacial processes include early cell adhesion, biofilm development, and the formation of passive layers on mineral surfaces (Safar et al., 2020). For example, microorganisms can catalyze metal accumulation through microfossilization, which is induced by microbe-metal interactions (Henne et al., 2021). When materials are immersed in seawater, a conditioning film rapidly forms on their surface. This film is a surface coating formed by the adsorption of biomolecules from the surrounding environment, altering the material’s inherent surface properties and facilitating microbial attachment (Bhagwat et al., 2021). Subsequently, microorganisms undergo the biofilm development process, which includes adhesion, intercellular interactions, proliferation, maturation, and dispersion (Costerton et al., 1999), as illustrated in Figure 4C (Anusha and Mulky, 2023). A deeper understanding of the interactions between microorganisms and metallic minerals requires analytical tools with high chemical sensitivity and spatial resolution. In recent years, the growing application of X-ray techniques, ranging from nanoscale to microscale, as well as large-scale synchrotron accelerators has provided new insights into microbial growth, mineral dissolution, redox transformations, and biomineralization processes (Templeton and Knowles, 2009).

The process by which bacteria adhere to mineral surfaces is not yet fully understood. Some studies suggest a relationship between bacterial cells and mineral surfaces. For example, *Pseudomonas aeruginosa* PAO1 tends to adhere to mineral surfaces using its flagella and/or pili. Surface roughness is a critical factor that may hinder attachment (Han M. et al., 2024). To better understand the underlying molecular processes and mechanisms, Yan et al. (2016) analyzed the adhesion of *Shewanella oneidensis* MR-1 to goethite using flow-cell attenuated total reflection (ATR), Fourier-transform infrared (FTIR) spectroscopy, combined with two-dimensional correlation spectroscopy (2D-COS) analysis. Other studies have described that interactions between goethite and bacteria can damage bacterial cells by piercing the cell wall, causing bacterial aggregation at the liquid-air interface Ma et al. (2017). Analyzing the physical interfacial properties between cell envelopes, outer membrane cytochromes responsible for interfacial electron transfer, and complex mineral particles remains challenging. Models explaining how microorganisms acquire electrons from solid donors are multifaceted, and include electron transfer via redox mediators such as H₂ or direct contact through membrane proteins (Liu J. et al., 2016; Mei et al., 2024). In recent years, molecular mechanisms and gene regulation have also become key areas of interest in studying microbe-mineral interactions. For instance, studies have shown that extracellular DNA promotes EET via pyocyanin in *P*. *aeruginosa* biofilms (Xiao et al., 2014; Saunders et al., 2020).

Microorganisms utilize EET and external minerals during their growth, a process accompanied by the conversion of chemical energy (Tian et al., 2019). Electroactive microorganisms can establish electrical contacts with other cells and minerals on their outer-surface or reduce soluble extracellular redox-active molecules such as flavins and humic substances. Cytochromes, conductive protein filaments, soluble electron shuttles, and abiotic conductive materials can significantly extend the electronic reach of microorganisms beyond the cell surface (Lovley and Holmes, 2021). Enzymes such as hydrogenases, which catalyze the oxidation of molecular hydrogen, can mediate electron transfer to metals and may facilitate the formation of minerals at the bacteria-mineral interface. Additionally, research has shown that some methanogens are electrogenic and corrosion-causing microbes. Many methanogens involved in MIC can thrive extensively at abiotic-biotic interfaces (Mei et al., 2024). Scheller et al. (2016) studied EET between ANME and sulfate reduction, they lent support to the hypothesis that interspecies extracellular electron transfer is the syntrophic mechanism for ANME.

### 4.3 MIC research in deep-sea environments

**Figure 5:**
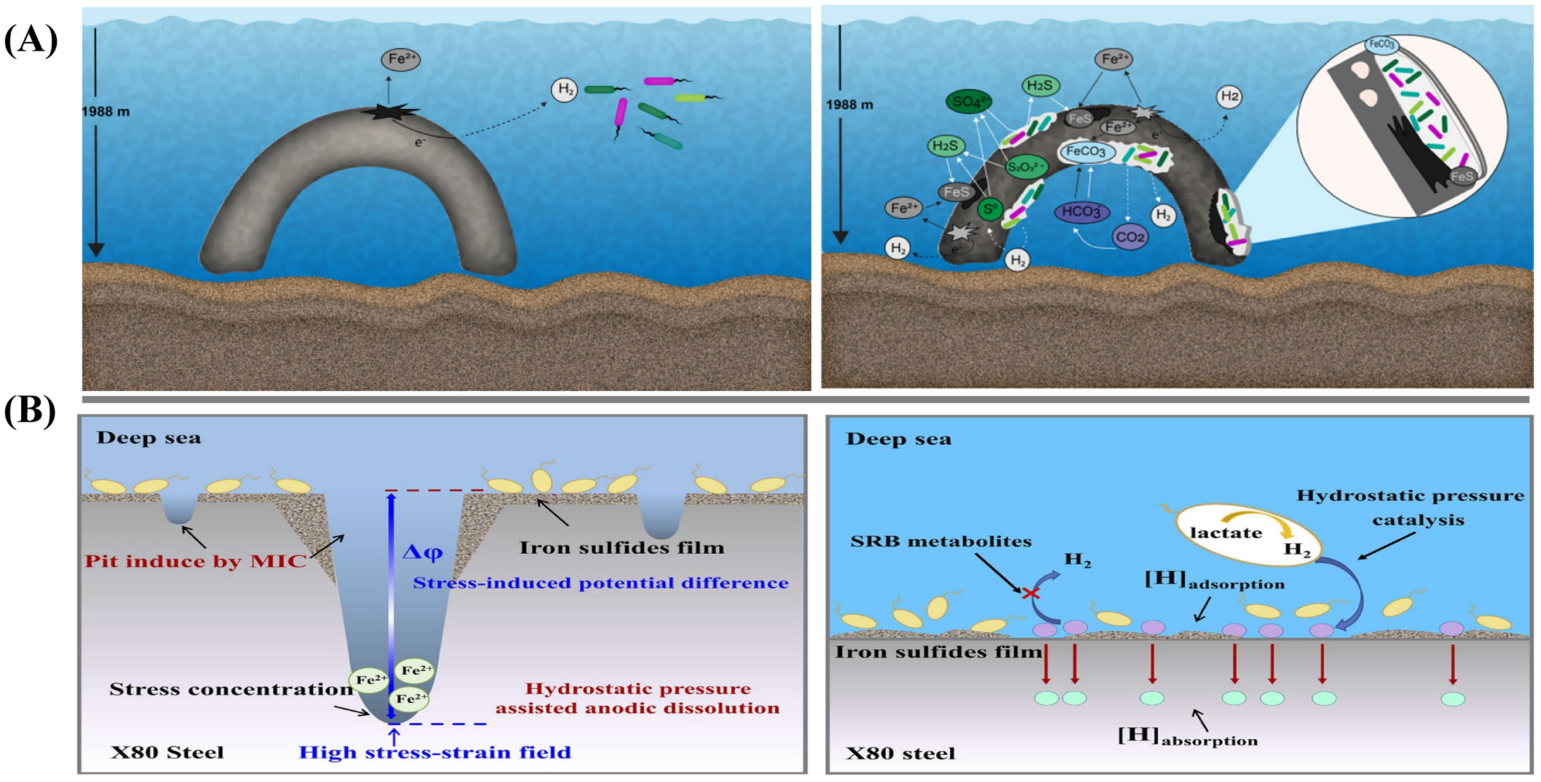
**(A)** Schematic model of abiotic (left panel) and biotic (right panel) processes inducing corrosion of steel on the mooring chain under deep-sea conditions (Rajala et al., 2022b); **(B)** Accelerated SCC mechanisms of X80 pipeline steel under the combined effects of SRB and hydrostatic pressure (Li J. et al., 2025).

Due to the unique environmental characteristics of the deep sea, such as high hydrostatic pressure and oligotrophic conditions, corrosion of materials in deep-sea environments differs from that in shallow seawater. Rajala et al. (2022b) conducted a deep-sea hanging plate experiment and detected significant MIC on the material surface after 10 years. Numerous microorganisms adhered to and extensively colonized the mooring chain surface, greatly accelerating the metal’s corrosion rate. The corrosion mechanism of mooring chains in deep-sea environments is illustrated in Figure 5A.

Currently, due to the challenges in simulating the extreme conditions of the deep sea, research on the impact of deep-sea environmental factors on microbial corrosion remains limited. Laboratory simulations must account for factors such as high hydrostatic pressure, oligotrophic conditions, seawater flow dynamics, and temperature. Li J. et al. (2025) investigated the accelerated SCC mechanism of X80 steel under the combined influence of SRP and hydrostatic pressure in a high-pressure reactor. By varying the hydrostatic pressure to simulate deep-sea conditions, they observed that hydrostatic pressure promoted MIC, as shown in Figure 5B. In this case, hydrostatic pressure and iron sulfide facilitated the hydrogen evolution reaction, further accelerating corrosion.

### 4.4 Corrosion data

Corrosion data are continuously collected across various industries, including petroleum, natural gas, shipping, and nuclear sectors, for risk assessment, component life prediction, and corrosion control (Li et al., 2015). Establishing and sharing corrosion data is an important topic at present. Multiple factors, such as material composition, microstructure, design parameters, as well as oxygen content, humidity, salinity, pH, temperature, and microbial communities, all influence the degradation of materials like pipelines. By integrating artificial intelligence, real-time corrosion monitoring can be achieved. For instance, Yao et al. (2019) proposed a method for detecting corrosion damage and other surface structural damages on hull structures based on convolutional neural networks. This method accelerates the application of artificial intelligence technologies in the field of shipbuilding and marine engineering, providing practical value for reasonably mitigating corrosion loss.

Currently, quantifiable data of MIC primarily manifest in corrosion weight loss and biofilm formation detection. Specifically, this includes corrosion rate, biofilm quantity, thickness, distribution, and the properties of corrosion products. Most theoretical and practical studies use methods such as SEM, CLSM, and electrochemical techniques to characterize biofilms and corrosion products. Additionally, many studies now employ various sensors, such as probes for the microenvironment within biofilms, to detect the microenvironment and determine their impact on biofilm-specific processes. For example, quantitative biofilm measurements and the detection of pH, polysaccharides, proteins, eDNA, and cytochrome C which is related to electron transfer mediators, are commonly used (Legner et al., 2020; Hunter Ryan and Beveridge Terry, 2005; Eddenden and Nitz, 2022; Jennings et al., 2015). The corrosion rate calculated using the formula shown below, where v_corr_ is the corrosion rate (mm/y), m_0_ is the initial weight of the coupon before weight loss testing (g), m_1_ is the weight of the coupon after testing and the corrosion products are removed (g). *K* is the unit constant (87,600), A is the working area of the coupon (cm^2^), t is the immersion period of the coupon (h), and *ρ* is the density of the coupon (g/cm^3^).


$$v_{\text{corr}}=\frac{(m_{0}-m_{1})\times K}{A\times t\times \rho }$$

## 5 MIC-biomineralization relationship in marine environments and prospects

The primary food source for deep-sea biota is the precipitation of particulate organic polymers (Dang et al., 2024). The progression from the formation of the conditioning film to the maturation of a biofilm on material surfaces can lead to MIC (Qian et al., 2022). Additionally, the transition from biofilm formation to the development of a biomineralized film can further influence MIC. In deep-sea environments microbial mineralization layers may either promote or inhibit corrosion. For example, the minerals formed through biomineralization can accelerate crevice corrosion and under-deposit corrosion.

**Figure 6:**
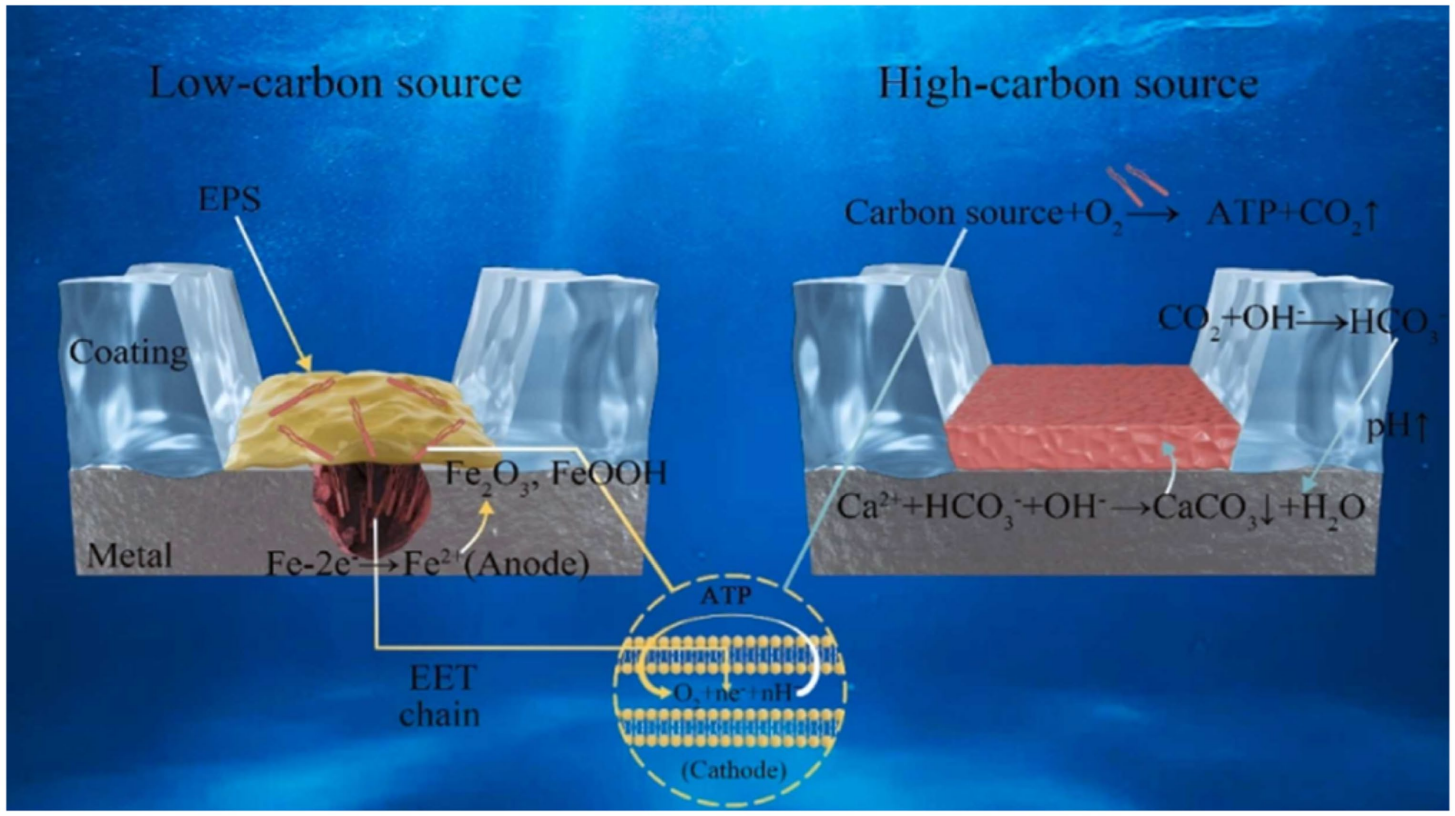
A schematic illustration describing the mechanism of the effect of carbon sources in media on biomineralization and corrosion processes (Hao et al., 2024).

The microbe-mineral interface is the primary site for biochemical MIC reactions, making its understanding crucial for deciphering MIC and biomineralization mechanisms. Under varying environmental conditions, such as different carbon source availability, the same microorganism may induce both corrosion and mineralization on the same material substratum, as illustrated in Figure 6. In addition, microorganisms can generate dense mineral films through biomineralization, which may exhibit protective characteristics and inhibit corrosion. Furthermore, interactions at the minerals-bacteria interface significantly influence microbial behavior (Gong et al., 2018). The stability of minerals and their morphologies, formed through mechanisms like EET at the microbe-material interface, is not always guaranteed. For instance, SRP can facilitate the transformation of metal oxides into metal sulfides, potentially accelerating corrosion (Duan et al., 2006). It is important to acknowledge that once corrosion occurs, the subsequent formation of a mineralized protective layer may not necessarily provide effective long-term protection (Mao et al., 2022).

### 5.1 Corrosion inhibition through biomineralization

The structure of biomineralized films plays a crucial role in determining the corrosion behavior of metals (Liu et al., 2022). The critical transition from biofilm to a biomineralized film is essential for ensuring long-term anti-corrosion properties, as it addresses the inherent instability of biofilm protection against corrosion (Liu et al., 2018). Microorganisms can induce the formation of biomineralized films, which may possess properties such as super hydrophobicity to reduce biological adhesion or enhanced density to achieve corrosion inhibition. Given the complex competitive, symbiotic, and cooperative interactions among different microorganisms, the synergistic effects of various microbial communities in corrosion inhibition have garnered significant attention.

#### 5.1.1 MIC and biomineralization in marine engineering cases

There have been some projects and experimental experiments exploring or applying biomineralization to suppress corrosion in relevant marine environments, especially in interesting works such as corrosion research on offshore structures, pipelines, and historical sunken ships.

In the study of historical sunken ships made of wood and steel materials, black corrosive substances have been commonly found. Zhao et al. (2024) discovered a distinct black corrosion pattern primarily concentrated in the interface region where barnacles attach to the wooden shipwrecks. This corrosion is mainly composed of FeS, FeS_2_, and Fe_3_S_4_, and exhibits a significant trend of extending inward along the surface of the wood. The origin of this black corrosion is intricately linked to barnacle cement, its role in biological corrosion, and the subsequent biomineralization process. The “black shell” formed on the gold coins found in the wreck of the SS Central America in 1857 played a key role in preserving them in an almost pristine state. In the years following the shipwreck, a large amount of steel on the wreck produced fine-grained iron mineral layers as geochemical precipitates on the coins. This coating served to protect the coins from future chemical or biological attacks (Melchiorre et al., 2019). The protection of a large number of cultural relics discovered in sunken ships is also an important issue, and many research works have been carried out (Pan et al., 2023; Yanagida et al., 2024; Grousset et al., 2016). In addition, a large number of studies have analyzed the attached microbial communities of underwater structures and conducted numerous studies on the mechanisms of single bacterial influence under laboratory conditions (Mugge et al., 2019; Lv et al., 2024b).

#### 5.1.2 Protective role of biomineralized layers on materials

**Figure 7:**
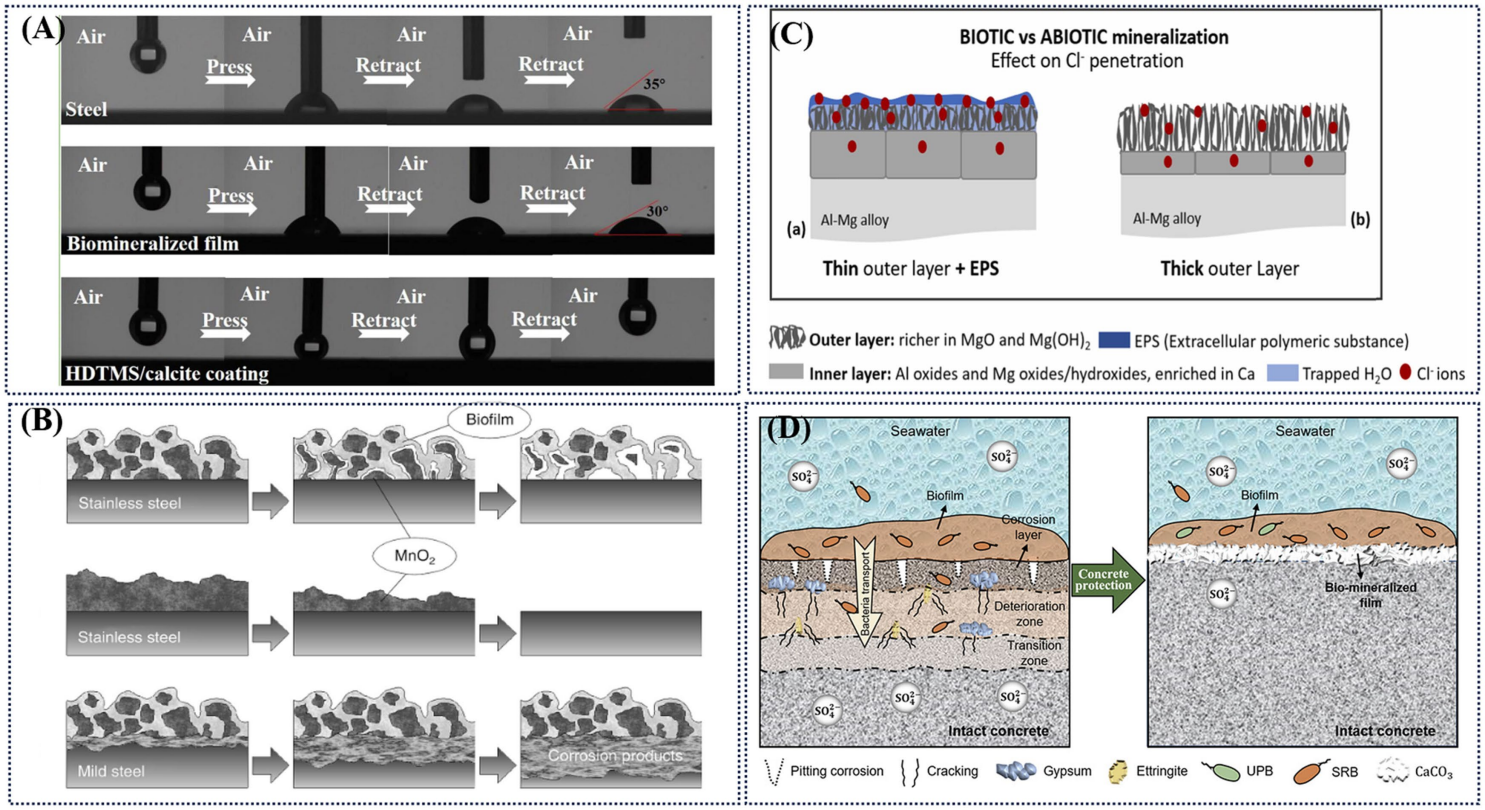
**(A)** Images of adhesion behavior of water droplets on steel, biomineralized film, and HDTMS/calcite coating (Zhang et al., 2022); **(B)** Corrosion products electrically insulated the biologically deposited minerals from the underlying metal (Olesen et al., 2000); **(C)** Cross-section schematic representation of Cl- ion penetration through surface layers on Al-Mg immersion in biotic/light side (a) and abiotic (b) conditions. **(D)** Biomineralization for corrosion inhibition (Sun et al., 2023).

Biomineralization products are primarily composed of inorganic–organic hybrid compounds. Compared to biofilms, the biomineralization layer formed between biofilms and metal ions exhibits higher stability and stronger barrier properties. Furthermore, alterations in biochemical parameters, microbial activity, and oxygen depletion collectively contribute to the protective function of the biomineralized layer. Biomineralization can provide protective effects by altering the surface properties of coatings, limiting the diffusion of dissolved oxygen, isolating corrosive ions in seawater. or disrupting electrical contact. Inspired by the nacre of seashells, Zhang et al. (2022) achieved the micro- or nanoscale surface roughness necessary for a superhydrophobic coating through *Bacillus subtilis*- induced mineralization, as shown in Figure 7A. Olesen et al. (2000) observed that when MnO₂ was directly bio-deposited on low-carbon steel, the accumulation of corrosion products on the steel surface hindered electrical contact between manganese oxide and the underlying metal, thereby preventing an increase in the corrosion rate, as shown in Figure 7B. Marques et al. (2024) indicates that under natural environmental conditions, the surface of Al-Mg alloys exposed to sunlight forms a mineralized layer composed of EPS and Ca-Mg, which hinders Cl^−^ and thereby protects the metal substrate, as shown in Figure 7C. Another study found that the biomineralization of Mn increased the open circuit potential of the 316 L stainless steel with deposited MnOOH and MnO_2_ (Shi et al., 2002). As discussed in Section 3.4.2, key biochemical parameters (e.g., pH) significantly influence the formation of the biomineralized layer. Using pH variation as an example, Guo et al. reported that synergistic biomineralization by *P*. *lipolytica* and *Bacillus subtilis* elevated the medium’s pH and induced carbonic anhydrase secretion, leading to the formation of a denser and more uniform biomineralized film (Guo N. et al., 2021). At the microbial level, while earlier studies hypothesized that aerobic and facultative microorganisms in the upper biomineralized layer could create localized anaerobic conditions favoring SRP growth, Wang et al. revealed a competitive relationship between mineralizing bacteria and SOB. Their findings showed that biomineralized film formation significantly reduced both the total and relative abundance of SRB communities and the proportion of SOB, while also suppressing functional genes involved in the sulfur cycle (Wang et al., 2025).

Consequently, protecting steel surfaces with biomineralized films requires a careful selection of bacteria capable of effective mineralization. Moreover, existing methods for mitigating metal biocorrosion are often costly and environmentally unfriendly. To address this problem, researchers have conducted have explored various strategies. Liu et al. (2018) proposed the formation of biofilms by the marine bacterium *P*. *lipolytica*, which subsequently developed into an organic–inorganic hybrid membrane. This hybrid membrane, consisting of multilayered calcite and EPS, demonstrated high and stable barrier protection efficiency while also providing in-situ self-healing activity. Lou et al. (2021b) investigated corrosion inhibition with Q235 carbon steel and found that *Shewanella putrefaciens* utilized its cell wall as nucleation site to induce the formation of a protective biomineralized layer containing calcite and extracellular polymers. This biomineralized layer exhibited wear resistance and could the ability to self-repair after mechanical damage. For other materials, such as Al alloys, Cu-Al alloys and Ti alloys, biomineralization has also been explored as a corrosion inhibition strategy. However, further research is required to elucidate additional mechanisms (Shen et al., 2020; George et al., 2016; Marques et al., 2024.; Zhu et al., 2020).

#### 5.1.3 Research on typical marine biomineralization and synergistic corrosion inhibition

SRP and methanogenic archaea have been extensively studied in the fields of corrosion and mineralization. Biomineralized films have been shown to reduce both the total amount and relative abundance of SRP. These films act as protective layers to control sulfate diffusion by isolating concrete from corrosive SRP communities, as shown in Figure 7D (Sun et al., 2023). Methanogenic archaea can also reduce iron from Fe (III) to Fe (II). Shang et al. (2020) demonstrated that in anaerobic environments rich in organic matter and Fe (III), *Methanosarcina barkeri* can similarly reduce Fe (III) to Fe (II) and Zerovalent Iron. This finding suggests significant potential applications in protecting iron materials from corrosion during sediment diagenesis.

Different bacterial groups, when present together, can exhibit synergistic mechanisms. Research on microbial communities in real-world environments can provide new perspectives for the future applications of biomineralization in natural marine and deep-sea settings. ANME frequently form cell consortia with SRP from the *Deltaproteobacteria* family. Chen et al. (2014) proposed a biomineralization mechanism mediated by anaerobic methane oxidation (AOM). They speculated that ANME cell consortia could interact with other microorganisms and their substrates via a silica shell. This silica accumulation mechanism contributes to the formation of clay minerals in marine sedimentary environments.

### 5.2 Promotion of corrosion by biomineralization

While the formation of biomineralized layers can inhibit uniform corrosion, it may also exacerbate localized corrosion processes such as pitting and crevice corrosion. For example, corrosion pits may form due to the presence of small anodes, and pitting corrosion can occur under the biomineralized films due to pigments secreted by microorganisms (Guo Z. et al., 2021; Liu et al., 2022). Interestingly, biomineralization can affect the visual appearance of corrosion by forming bubbles, these may create ecological niches for microorganisms that ultimately contribute to MIC (Martinez et al., 2022). Additionally, as described in Section 5, microbial-material interfacial interactions can lead to instability in mineral formation and their morphology, potentially promote corrosion.

### 5.3 Emerging technologies

Researchers can currently construct strains with enhanced mineralization and extracellular polymer secretion capabilities through microbial genetic engineering, thereby promoting the formation of denser, more uniform mineralized layers on metal surfaces to inhibit corrosion. Alternatively, through biofilm management technology, the porous, highly adhesive, and low-cost characteristics of EPS can be combined with existing corrosion inhibitors to develop multifunctional, versatile bio-coatings that sustainably enhance metal corrosion resistance. Li Z. et al. (2025) genetically engineered a mutant strain (*Shewanella oneidensis* PCA) capable of overproducing the electron shuttle phenazine-1-carboxylic acid (PCA). By integrating high-resolution passivation film characterization with molecular biology techniques, they systematically elucidated a novel microbial corrosion mechanism involving synergistic direct–indirect electron transfer that accelerates passive film dissolution. This work highlights the dual significance of microbial genetic engineering in both fundamental research and practical applications. Li X. et al. (2025) first utilized the biofilm formed by corrosion-protective microorganisms (*Tenacibaculum mesophilum* D-6) as a scaffold to construct a novel “smooth liquid-injected porous surface” (SLIPS) coating (TM@SLIPS). The biofilm’s high adhesion, flexibility, and porous structure provide an ideal framework for the uniform distribution of lubricants, thereby enhancing the coating’s durability. It is produced using bio-based materials through controlled cultivation, enabling scalable production at low cost and easy scalability.

### 5.4 Environmental and ethical considerations of deep-sea MIC and biomineralization processes

Corrosion is not only related to economic losses, but its impact on the environment should also not be underestimated. Over 3,800 World War II sunken ships containing thousands of tons of oil are at risk of structural collapse due to continuous corrosion, posing a threat to the marine environment (Carter et al., 2021). As deep-sea engineering and exploration advance, the environmental consequences of MIC and biomineralization have become increasingly relevant. These microbial processes, while naturally occurring, can be exacerbated by anthropogenic materials and interventions, leading to long-term disruptions of fragile and poorly understood ecosystems. This section explores key areas of concern—including microbial biodiversity, toxic byproducts, long-term ecological consequences, mitigation strategies, and ethical considerations—and outlines approaches to reduce ecological impact while promoting sustainable deep-sea operations.

#### 5.4.1 Impacts on biodiversity and community structure

MIC and biomineralization processes can significantly reshape microbial community structures by creating chemically selective microenvironments on metal surfaces. These conditions often favor metabolically aggressive groups such as SRP, methanogens, and IOP, which may displace native, slow-growing microbial taxa. The loss of microbial diversity and functional redundancy can impair key ecosystem services such as methane oxidation, nitrogen cycling, and trophic support for benthic macrofauna (Shu and Huang, 2022).

To reduce these impacts, several mitigation strategies can be employed. Material selection plays a crucial role: inert substrates such as titanium alloys or ceramics discourage biofilm formation, while surface modification techniques—like nanopatterning or antifouling coatings—can further inhibit microbial adhesion. Another approach involves preconditioning surfaces with benign microbial consortia to outcompete corrosive taxa, though care must be taken to avoid non-native species introduction. Site selection also matters; locating infrastructure away from ecologically sensitive areas such as cold seeps or hydrothermal vents minimizes disturbance. Finally, building microbial baseline datasets and performing community-level monitoring allow researchers to detect shifts in biodiversity and assess the ecological footprint of engineering activities.

#### 5.4.2 Toxic byproducts and metal mobilization

The metabolic byproducts of MIC, including hydrogen sulfide, organic acids, and ammonia, are not only corrosive but can be toxic to surrounding organisms. In confined environments such as crevices or sediment-buried structures, these compounds may accumulate to harmful levels. At the same time, microbial activity can enhance the mobilization of metal ions (Fe^2+^, Mn^2+^, Ni^2+^, Cr^3+^) from structural materials, with potentially toxic effects due to their high bioavailability in nanoparticulate or reduced forms (Mallick et al., 2025).

Mitigating these effects requires a multi-pronged approach. Corrosion-resistant materials, such as high-alloy stainless steels with molybdenum or titanium, can limit metal ion release. Where biomineralization is used intentionally, controlling the precipitation of low-solubility minerals like calcite or magnetite under stable geochemical conditions helps immobilize harmful metals. The addition of stabilizing agents (e.g., phosphate, silicate) may enhance long-term mineral stability. *In situ* monitoring systems—including pH sensors, redox probes, an d electrochemical detectors—should be integrated to detect toxic compound buildup in real time, enabling timely responses. Hydrodynamic design considerations, such as ensuring adequate current flow, can also help disperse harmful byproducts, reducing localized toxicity.

#### 5.4.3 Long-term environmental consequences

Deep-sea ecosystems are characterized by low metabolic rates and long recovery times. Persistent MIC and biomineralization processes can irreversibly alter substrate characteristics, disrupt microbial recolonization, and reduce habitat suitability for benthic species. Accumulated mineral layers or biofilms can inhibit larval settlement or shift faunal community structures. Over time, these changes may shift ecological baselines and suppress resilience to other anthropogenic pressures, such as mining or climate-driven changes (Levin et al., 2016).

To prevent long-term degradation, engineering solutions must incorporate predictive models of corrosion and material aging to anticipate the timing and severity of biological interactions. Substrate designs should favor modular components that can be maintained, cleaned, or replaced to prevent chronic ecological disruption. Where disturbance has occurred, pilot efforts in ecological rehabilitation—such as reseeding native microbes or deploying biodegradable structures—can help initiate recovery, though such approaches remain experimental.

#### 5.4.4 Mitigation and monitoring strategies

Sustaining ecological balance in deep-sea environments affected by MIC and biomineralization requires systemic, lifecycle-based strategies. Because infrastructure often remains on the seafloor for decades, proactive environmental integration is vital. First, predictive corrosion modeling under high-pressure, low-temperature conditions should inform design and material selection, minimizing long-term reactivity and emissions. Second, dynamic environmental monitoring—using autonomous platforms equipped with microbial samplers, chemical sensors, and imaging systems—should track both corrosion processes and ecological responses.

Importantly, these monitoring systems should not only serve engineering goals but also detect shifts in ecosystem health, such as biodiversity loss, metabolite accumulation, or habitat degradation. Feedback from this data must guide adaptive management actions, including component replacement, intervention pause, or spatial redesign of deployments. In cases of severe or cumulative impact, localized remediation—such as surface neutralization or selective module recovery—may be warranted. Moreover, international protocols for post-deployment ecological assessment and data transparency will be essential, particularly in regions beyond national jurisdiction.

Ultimately, integrating engineering resilience with ecological foresight will be key to minimizing long-term environmental harm and promoting a dynamic balance between technological use and deep-sea ecosystem function.

#### 5.4.5 Ethical considerations in microbial interventions

The application of microbial approaches for deep-sea corrosion control—such as engineered biomineralization or microbial inhibition—raises significant ethical considerations. Deep-sea ecosystems remain among the most poorly understood on the planet, and human interventions may carry unpredictable or potentially irreversible consequences. Actions like introducing microbial consortia, altering substrate chemistry, or influencing microbial succession risk disturbing critical ecological processes or destabilizing host–microbe interactions.

Ethical practice in this context requires strict adherence to the precautionary principle, supported by comprehensive risk assessments and sustained monitoring both prior to and following any intervention. In regions beyond national jurisdiction, where governance is limited, international cooperation, transparent data sharing, and adherence to environmental impact protocols are particularly vital. Above all, ethical stewardship demands that deep-sea microbiomes be regarded not merely as tools for engineering solutions but as vital components of delicate, interconnected ecosystems that warrant respect and protection.

## 6 Conclusion

Deep-sea MIC and biomineralization is an interdisciplinary field that integrates materials science, microbiology, geochemistry, and oceanography. Multidisciplinary approaches and collaborative research teams are essential for understanding how individual microbial cells and microbial communities interact with their surroundings on the nanoscale or microscale. Emerging methods and technologies, such as bio-electrochemostasis for exploring the impact of flow dynamics on corrosive biofilms under simulated deep-sea high-pressure conditions (Ivanovich et al., 2025), are needed to characterize and elucidate molecular-level interactions, including bonding, electron transfer, and various physiological activities at the microbial-material interface. Advanced techniques, including multi-omics analysis, machine learning, bioinformatics, gene editing, real-time spectroscopy and computational simulations, can help identify key microbial drivers in real-world environments and explain their mechanisms of action. Investigating the mechanisms of deep-sea corrosion and biomineralization can lead to sustainable strategies for corrosion prevention in deep-sea settings.
